# Characterization of a Novel LUCAT1/miR-4316/VEGF-A Axis in Metastasis and Glycolysis of Lung Adenocarcinoma

**DOI:** 10.3389/fcell.2022.833579

**Published:** 2022-05-13

**Authors:** Lishui Wang, Yan Xie, Jing Wang, Ying Zhang, Shibiao Liu, Yao Zhan, Yinghui Zhao, Juan Li, Peilong Li, Chuanxin Wang

**Affiliations:** ^1^ Department of Clinical Laboratory, Qilu Hospital, Cheeloo College of Medicine, Shandong University, Jinan, China; ^2^ Department of Clinical Laboratory, The Second Hospital, Cheeloo College of Medicine, Shandong University, Jinan, China; ^3^ Shandong Engineering & Technology Research Center for Tumor Marker Detection, Jinan, China; ^4^ Shandong Provincial Clinical Medicine Research Center for Clinical Laboratory, Jinan, China; ^5^ Shandong Technology Innovation Center for Big Data and Precision Medicine of Cancer, Jinan, China

**Keywords:** lung adenocarcinoma, exosomes, lncRNA LUCAT1, mir-4316, metastasis, glycolysis

## Abstract

**Objective:** Accumulating literatures suggested that long non-coding RNAs (lncRNAs) were involved in tumorigenesis and cancer progression in lung adenocarcinoma (LUAD). However, the precise regulatory mechanism of lncRNA Lung cancer-associated transcript 1 (LUCAT1) in LUAD is not well defined. In this study, we aimed to investigate the biological function and mechanism of lncRNA LUCAT1 in regulating tumor migration and glycolysis of LUAD.

**Methods:** High throughput sequencing was performed to identify differentially expressed lncRNAs between LUAD patients and healthy controls. The expression levels of LUCAT1 in LUAD clinical specimens or cell lines were evaluated by *In situ* hybridization (ISH) and quantitative Real-Time Polymerase Chain Reaction (qRT-PCR). Functional experiments, including wound-healing, transwell invasion assays, glucose absorption, lactate metabolism and tumor xenograft experiments were conducted to identify the biological functions of LUCAT1 in LUAD. Silencing of LUCAT1, over-expression of LUCAT1 and miR-4316 were generated in LUAD cell lines to verify the regulatory mode of LUCAT1-mir-4316-VEGFA axis.

**Results:** Our findings revealed that lncRNA LUCAT1 was significantly up-regulated in LUAD serum exosomes, tumor tissues, and LUAD cells in comparison with corresponding controls. Receiver operating characteristic curve (ROC) analysis indicated that the area under the curve (AUC) value of serum exosomal LUCAT1 reached 0.852 in distinguishing LUAD patients from healthy individuals. High expression of LUCAT1 in LUAD patient tissues was associated with enhanced Lymph Node Metastasis (LNM), advanced Tumor Node Metastasis (TNM) stage and poorer clinical outcome in LUAD patients. Knockdown of LUCAT1 inhibited LUAD cell metastasis and glycolysis *in vitro* as well as tumor metastasis *in vivo*, while overexpression of LUCAT1 induced a promoted LUAD metastasis and glycolysis. Furthermore, mechanistic investigations revealed that LUCAT1 elevated LUAD cell metastasis and glycolysis by sponging miR-4316, which further led to the upregulation of VEGFA. Finally, the regulatory axis LUCAT1-miR-4316-VEGFA was verified in LUAD.

**Conclusion:** Our present research suggested that LUCAT1 facilitate LUAD cell metastasis and glycolysis *via* serving as a competing endogenous RNA to regulate miR-4316/VEGFA axis, which provided a novel diagnostic marker and therapeutic target for LUAD patients.

## Introduction

Lung cancer was the most common cause of cancer-related death in the world and the 5-year survival rate was about 15% (Torre et al., 2015; Jia et al., 2019). Non-small cell lung carcinoma (NSCLC) accounts for 85% of all lung cancer cases, approximately half of NSCLC cases are lung adenocarcinoma (LUAD), which arises from type Ⅱ alveolar cells in the small airway epithelial (Travis et al., 2015; Denisenko et al., 2018). Metastasis and relapse were two main causes of death of patients with LUAD (Li et al., 2016). Although there have been some significant advances in chemotherapy, radiotherapy and immunotherapy, the 5-year survival rate was still unsatisfactory (Hirsch et al., 2017). Hence, there was an urgent need to illustrate the underlying mechanisms of occurrence and development of LUAD and explore potential diagnostic and prognostic biomarkers for LUAD.

Long noncoding RNAs (lncRNAs) were a class of special RNA molecular that are more than 200 nucleotides in length and have extremely limited capacity of protein-coding (Mercer et al., 2009; Ponting et al., 2009). As one of typical modes of epigenetic regulation, lncRNAs were involved in various diseases, including cancer. Several studies have demonstrated that lncRNAs played pivotal roles in a variety of biological processes, such as cellular growth, proliferation, differentiation, migration, and invasion of cancer cells (Kretz et al., 2013; Zhang S. et al., 2019; Wang et al., 2019). The underlying regulatory mechanisms of lncRNAs were diverse, they may participate in the regulation of protein-DNA and protein-protein interactions, or directly bind to mircoRNAs and proteins as decoys, and thus modulate gene expression at transcriptional or post-transcriptional level (Tian et al., 2020). As competitive endogenous RNAs (ceRNAs), lncRNAs played vital roles in the lncRNA-microRNA-mRNA regulatory network of gene expression (Salmena et al., 2011). In addition, previous studies have shown that some aberrantly expressed lncRNAs were associated with occurrence and development of malignant tumors *via* regulation of glycolysis, including colorectal cancer, liver cancer, pancreatic cancer, etc. For example, LncRNA HULC directly binds to two glycolytic enzymes, LDHA, and PKM2, leading to elevated phosphorylation of these two enzymes and consequently speeding up glycolysis in liver cancer (Wang et al., 2020). In pancreatic cancer, LINC00261 promotes c-myc-mediated aerobic glycolysis by activating miR-222-3p/HIPK2/ERK axis and sequestering IGF2BP1, suggesting an essential role of lncRNAs in cell glycolysis (Zhai et al., 2021). Unlike normal cells, cancer cells have a special phonotype of glucose metabolism. Although there was plenty of oxygen, cancer cells tended to produce energy by glycolysis in the cytosol with production of lactic acid, which termed as “aerobic glycolysis” (Helmlinger et al., 2002; Elf and Chen, 2014). Aerobic glycolysis can rapidly provide energy for cancer cells to proliferate and survive. However, there are still a lot of gaps need to explore about lncRNAs which mediated aerobic glycolysis involved in the occurrence and development of LUAD.

Lung cancer-associated transcript 1 (LUCAT1), also identified as smoke and cancer associated lncRNA1 (SCAL1), was a novel lncRNA which first discovered in smoking-related lung cancer (Zhang L. et al., 2019; Liu et al., 2020). Compared with normal tissues, LUCAT1 was highly upregulated in NSCLC tissues. Usually, USCLC patients with higher LUCAT1 level tend to have poorer prognosis (Zheng A. et al., 2019). Evidence has shown that LUCAT1 could participate in proliferation, migration and invasion of esophageal squamous cell carcinoma (Yoon et al., 2018), ovarian cancer (Yu et al., 2018), prostate cancer (Liu et al., 2020), et al. Nevertheless, whether LUCAT1 influences metastasis and glycolysis in LUAD, and if yes, what is the regulatory mechanism, remains to be further explored. VEGF-A was the most potent pro-angiogenic factor that directly induced endothelial cell proliferation, migration and promotes tumor growth (Claesson-Welsh and Welsh, 2013). It has been reported that miR-4316 inhibits gastric cancer proliferation and migration *via* directly targeting VEGF-A. However, it is unclear whether miR-4316 regulate the metastasis of LUAD through VEGF-A (Krawczyk et al., 2017).

In this study, we verified that LUCAT1 was significantly upregulated in LUAD, and enhanced LUCAT1 promoted migration and invasion both *in vitro and in vivo*. In addition, LUCAT1 accelerated aerobic glycolysis. Mechanistically, LUCAT1 acted as miRNA sponge to absorb miR-4316, and thus releasing VEGFA. Therefore, the uncovering of LUCAT1/miR-4316/VEGFA axis in LUAD may provide theoretical support for finding novel diagnostic biomarkers and therapeutic targets of LUAD patients.

## Results

### Lung Cancer-Associated Transcript 1 Was Upregulated in Lung Adenocarcinoma Serum Exosomal Samples and May Serve as a Biomarker

**FIGURE 1:**
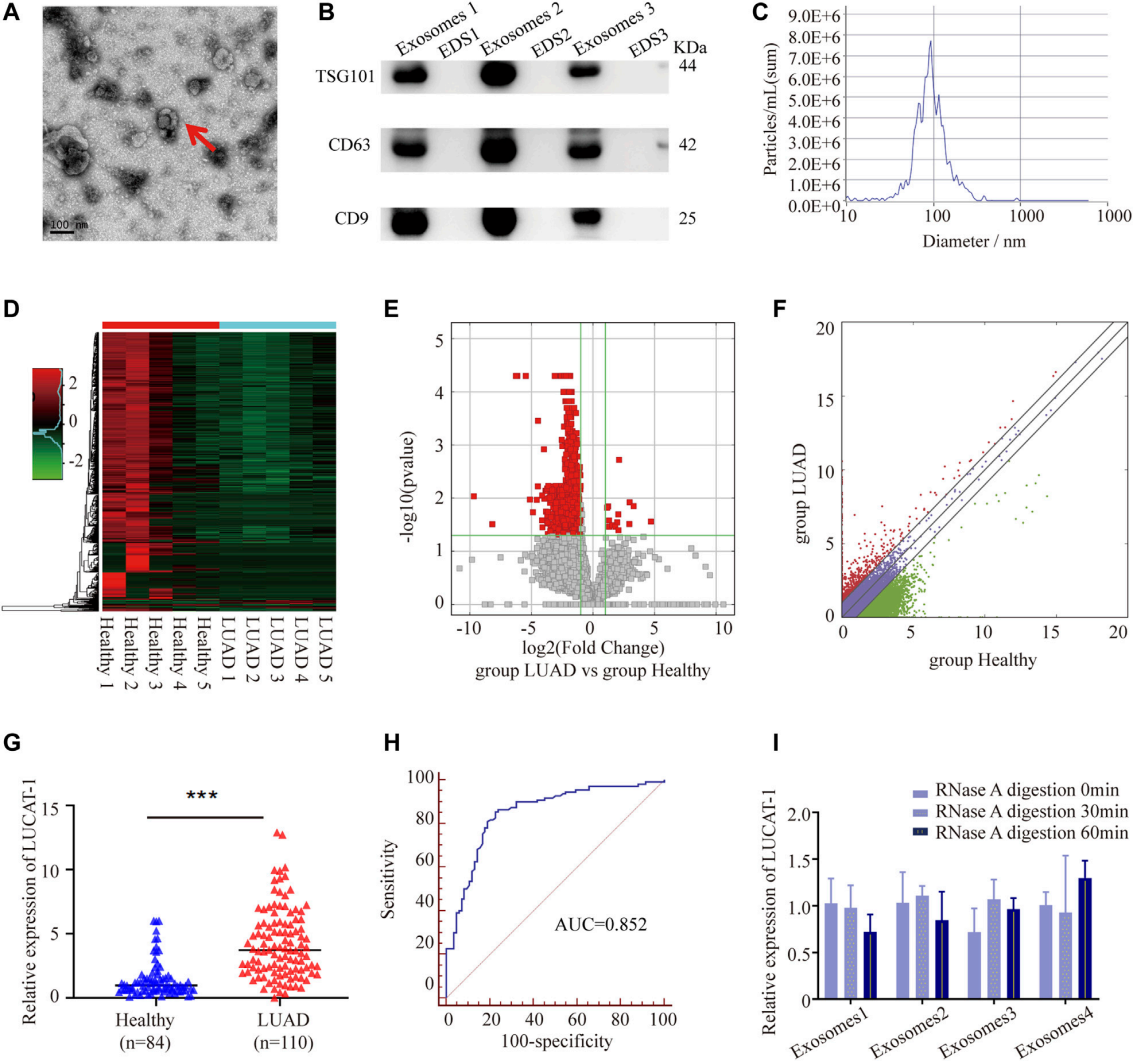
Tumor-originated exosomal LUCAT1 as a circulating biomarker for LUAD. **(A)** Transmission electron microscopy images of exosomes. Scale bar: 100 nm. **(B)** Western blot images of exosomal markers (TSG101, CD63, CD9). **(C)** Size distribution and concentration of serum exosomes. Heatmap **(D)**, volcano plot **(E)** and scatter plot **(F)** showed the differentially expressed serum exosomal lncRNAs from the high-throughput sequencing analysis. **(G)** Comparison of serum exosomal LUCAT1 expression between LUAD patients and healthy controls detected by qRT-PCR. **(H)** ROC curves for the determination of the diagnostic performance of serum exosomal LUCAT1. **(I)** Comparison of the expression level of LUCAT1 before and after RNase A treatment. *p* values were calculated using two-sided paired t-test (*p < 0.05; **p < 0.01; ***p < 0.001).

Exosomal samples isolated from LUAD patients and healthy controls were validated by performing Transmission electron microscopy (TEM), western blotting (WB) and nanoparticle tracking analysis (NTA). As shown, these vesicles showed typical oval-shaped extracellular vesicles with diameters of 50–150 nm by TEM (Figure 1A). WB demonstrated that the concentrations of the exosome surface protein markers, TSG101, CD63, and CD9, were enriched in serum exosomes but not in exosome-depleted supernatants (EDS) (Figure 1B). NTA showed that the diameters of most serum exosomes were mainly 91.6 nm (Figure 1C). All these results suggested that we successfully purified exosomes from the serum, which laid a foundation for further analysis.

High throughput sequencing was performed using 50 LUAD serum exosomal samples and 50 healthy controls (the 50 LUAD and 50 healthy samples were grouped and mixed into 5 repeats, respectively). The results showed that a total of 722 differentially expressed lncRNAs between the two groups were identified according to our screening disciplines, including 14 up-regulated and 708 down-regulated lncRNAs. Cluster analysis heat map, scatter plot, and volcano plot were shown to present the distinguishable lncRNAs expression profiles (Figures 1D–F).

Since up-regulated lncRNAs have greater potential to be used as early diagnostic markers or therapeutic targets, we focused on 7 most upregulated lncRNAs, including ENST00000417930, ENST00000578785, TCONS_l2_00006336, uc010jub.1, ENST00000569809, ENST00000430694, and ENST00000513626, among which, only ENST00000417930, uc010jub.1 and ENST00000513626 were successfully designed with specific primers. To confirm the High throughput sequencing results, expression levels of three serum exosomal lncRNAs were measured in 84 LUAD patients and 110 healthy controls through qRT-PCR, the results showed that only ENST00000513626 (LUCAT1) was significantly up-regulated in LUAD patients (Figure 1G), while the other two genes were not detectable due to low expression concentrations. The potential diagnostic value of serum exosomal LUCAT1 was evaluated through Receiver operating characteristic curve (ROC) analysis. As expected, the area under the ROC curve (AUC) of serum exosomal LUCAT1 for LUAD diagnosis was 0.852 (95% CI = 0.794–0.898), with a diagnostic sensitivity and specificity reaching 85.45 and 77.38%, respectively (Figure 1H). These data suggested that LUCAT1 was a promising biomarker for the diagnosis of LUAD and may play an important role in LUAD progression.

VEGF-A was the most potent pro-angiogenic factor that directly induced endothelial cell proliferation, migration and promotes tumor growth. It has been reported that miR-4316 inhibits gastric cancer proliferation and migration *via* directly targeting VEGF-A. However, it is unclear whether miR-4316 regulate the metastasis of LUAD through VEGF-A.

The instability of majority of the lncRNAs in serum remains a significant limitation for clinical application. Serum exosomes samples were incubated with RNase A for 0, 30, and 60 min, and then exosomal LUCAT1 expression was measured. Interestingly, the expression levels of exosomal LUCAT1 remained unchanged upon RNase A treatment (Figure 1I), which fully proved its sufficient suitability as tumor markers for LUAD diagnosis.

### Lung Cancer-Associated Transcript 1 Was Significantly Up-Regulated in Lung Adenocarcinoma Tissues and Cell Lines

**FIGURE 2:**
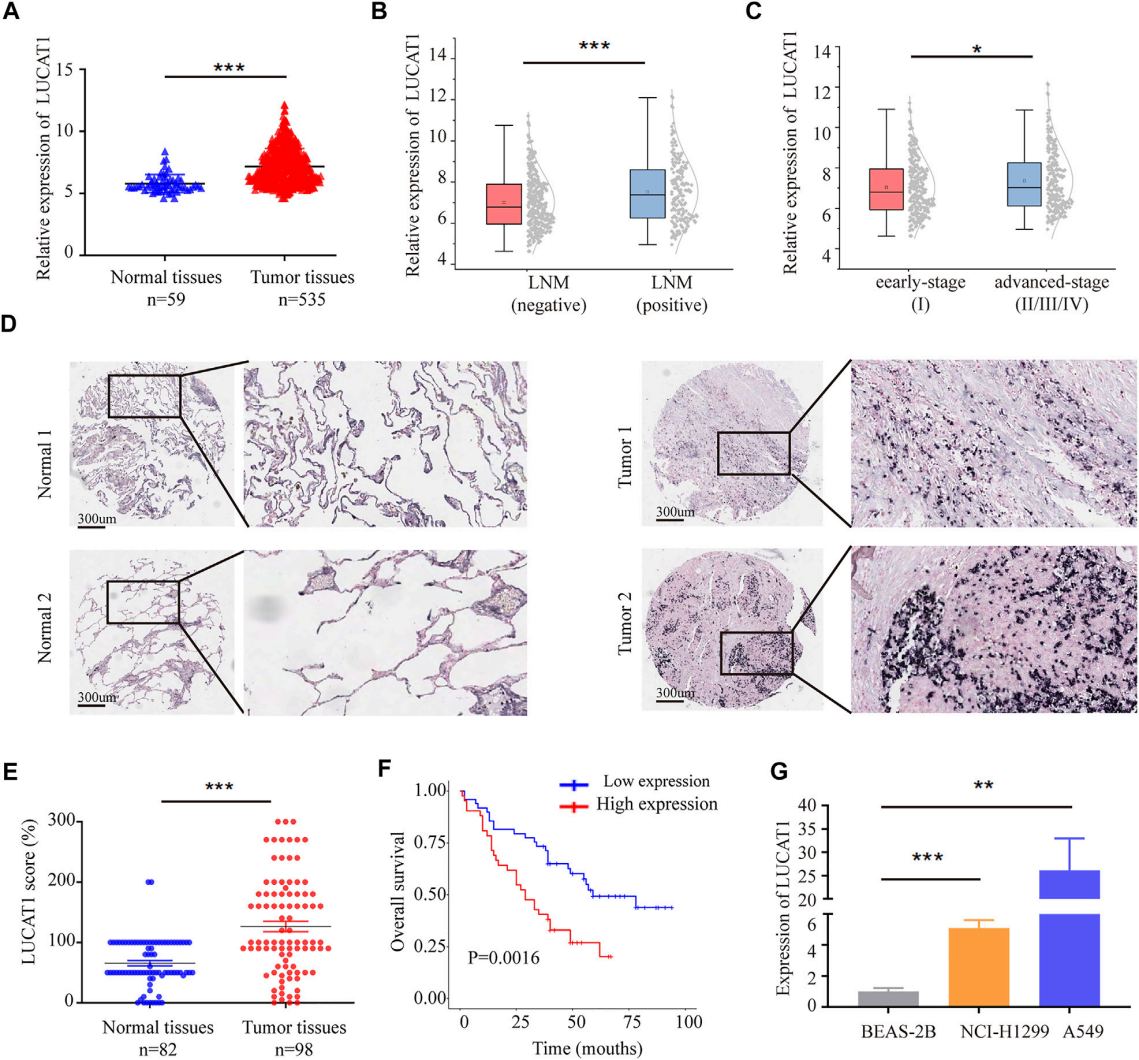
LUCAT1 was upregulated in LUAD tissues, and its overexpression correlates with poor outcome of LUAD. **(A)** Relative expression of LUCAT1 in LUAD compared with normal tissues was analysed by using TCGA datasets. **(B)** Relative expression level of LUCAT1 in LUAD with lymph node metastasis and non-lymph node metastasis. **(C)** Expression level of LUCAT1 in early-stage LUAD patients (stage I) with advanced-stage LUAD patients (stage II/III/IV). **(D,E)** ISH analysis of LUCAT1 expression in LUAD tissues (*n* = 98) and normal tissues (*n* = 82), Scale bar: 300 µm. **(F)** Kaplan-Meier analysis for overall survival of LUAD patients with low and high LUCAT1 expression (*p* = 0.0016). **(G)** qRT-PCR was performed to detect the expression of LUCAT1 in normal lung epithelial cells and LUAD cells. A two-tailed student’s t-test was used to analyse the comparison between the two groups (*p < 0.05; **p < 0.01; ***p < 0.001).

We also analyzed the expression data of LUCAT1 in 535 LUAD tissues and 59 normal tissues from The Cancer Genome Atlas (TCGA) database. LUCAT1 was significantly up-regulated in LUAD tumors than in non-tumor tissues (Figure 2A). Analyzing of clinical data revealed that LUCAT1 expression level was positively correlated with higher rates of Lymph Node Metastasis (LNM) (Figure 2B) and advanced Tumor Node Metastasis (TNM) stage (Figure 2C).

To further test whether expression level of LUCAT1 correlated with the outcomes of LUAD patients, the expression level of LUCAT1 was detected in 98 LUAD patients with different clinicopathological features and 82 paired normal tissues using *In situ* Hybridization (ISH). Consistent with TCGA results, LUCAT1 expression level was higher in LUAD tissues compared with normal tissues (Figures 2D,E). Kaplan-Meier survival analysis revealed that patients with high LUCAT1 expression had a markedly lower overall survival compared to those with low LUCAT1 expression (Figure 2F).

To understand the role of LUCAT1 in LUAD cells, the expression of LUCAT1 was detected in normal lung epithelial cells (BEAS-2B) and LUAD cells (NCI-H1299 and A549) by qRT-PCR analysis. As shown in Figure 2G, LUCAT1 expression in NCI-H1299 and A549 cells was much higher than that in BEAS-2B cells. Taken together, all these results indicated that LUCAT1 was significantly overexpressed in LUAD and might be a potential biomarker for predicting prognosis in LUAD patients.

### Lung Cancer-Associated Transcript 1 Was Required for Efficient Metastasis of Lung Adenocarcinoma *in vitro*

**FIGURE 3:**
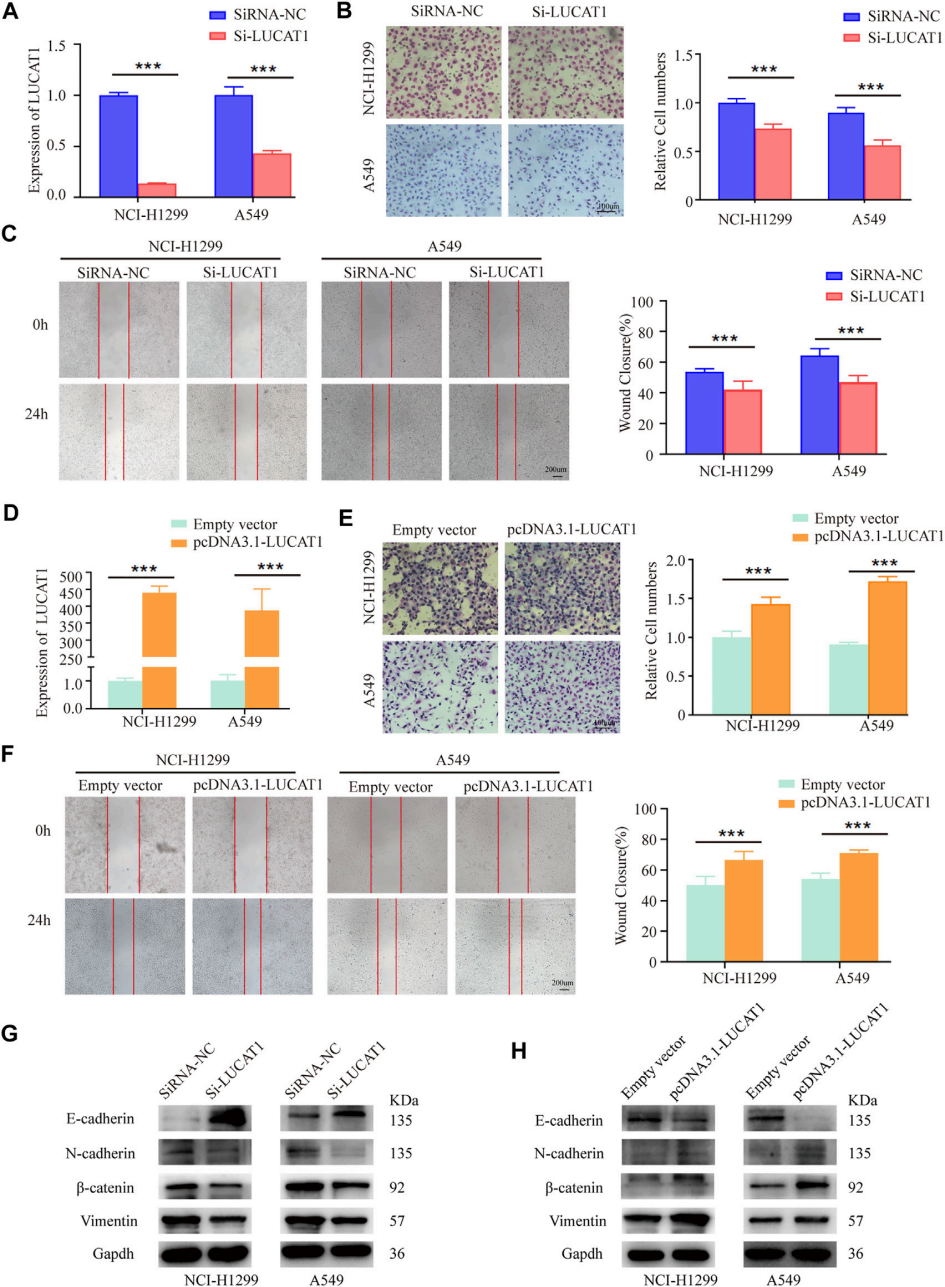
LUCAT1 promotes migration and invasion of LUAD cells *in vitro*. **(A)** LUCAT1 silencing efficiency of NCI-H1299 and A549 cells were verified by qRT-PCR analysis. **(B)** Transwell assay for investigating invasion change in NCI-H1299 and A549 cells after LUCAT1 knockdown, Scale bar: 100 µm. **(C)** The migration of LUCAT1-silenced LUAD cells were detected by wound healing assay. Scale bar: 200 µm. **(D)** The relative mRNA levels of LUCAT1 in LUAD cells transfected with pcDNA3.1- LUCAT1, or control. **(E)** Transwell assay were performed to determine the invasion change in NCI-H1299 and A549 cells upon LUCAT1 was overexpressed. Scale bar: 100 µm. **(F)** The migration of LUCAT1-overexpression LUAD cells were detected by wound healing assay. Scale bar: 200 µm. **(G,H)** The relative expression levels of E-cadherin and vimentin were determined by western blot in NCI-H1299 and A549 cells with LUCAT1 knockdown. Comparisons between two groups were analysed using two-sided paired t-test (*p < 0.05; **p < 0.01; ***p < 0.001).

In order to better investigate the biological functions of LUCAT1 in the tumorigenesis and development of LUAD, loss- and gain-of function experiments were conducted in NCI-H1299 and A549 cells. Specific siRNAs were used to knock down LUCAT1 expression, the knockdown efficiency of LUCAT1 in two LUAD cell lines were verified by qRT-PCR (Figure 3A). As shown, LUCAT1 knockdown strikingly attenuated the invasive abilities of NCI-H1299 and A549 cells (Figure 3B). Similarly, the migration capability of NCI-H1299 and A549 cells in wound healing assays was significantly decreased after the silence of LUCAT1 (Figure 3C). Subsequently, NCI-H1299 and A549 cells were transfected with the overexpression lentiviral vector (pcDNA3.1- LUCAT1) (Figure 3D). The adverse results were obtained, that is, overexpressed LUCAT1 strikingly enhanced the invasive abilities of two LUAD cells (Figure 3E). Additionally, wound healing assay showed that LUCAT1 overexpression significantly promoted the migratory ability of NCI-H1299 and A549 cells (Figure 3F).

Also, WB analysis revealed that LUCAT1 silencing could induce a significant elevation in epithelial maker E-cadherin expression, and a decreased expression of mesenchymal makers, including N-cadherin, β-catenin, and vimentin (Figure 3G). Conversely, LUCAT1 overexpression significantly increased mesenchymal makers N-cadherin, β-catenin, and vimentin expression, and decreased the expression of epithelial protein E-cadherin (Figure 3H).

### Lung Cancer-Associated Transcript 1 Knockdown Repressed Lung Adenocarcinoma Cell Metastasis *in vivo*

**FIGURE 4:**
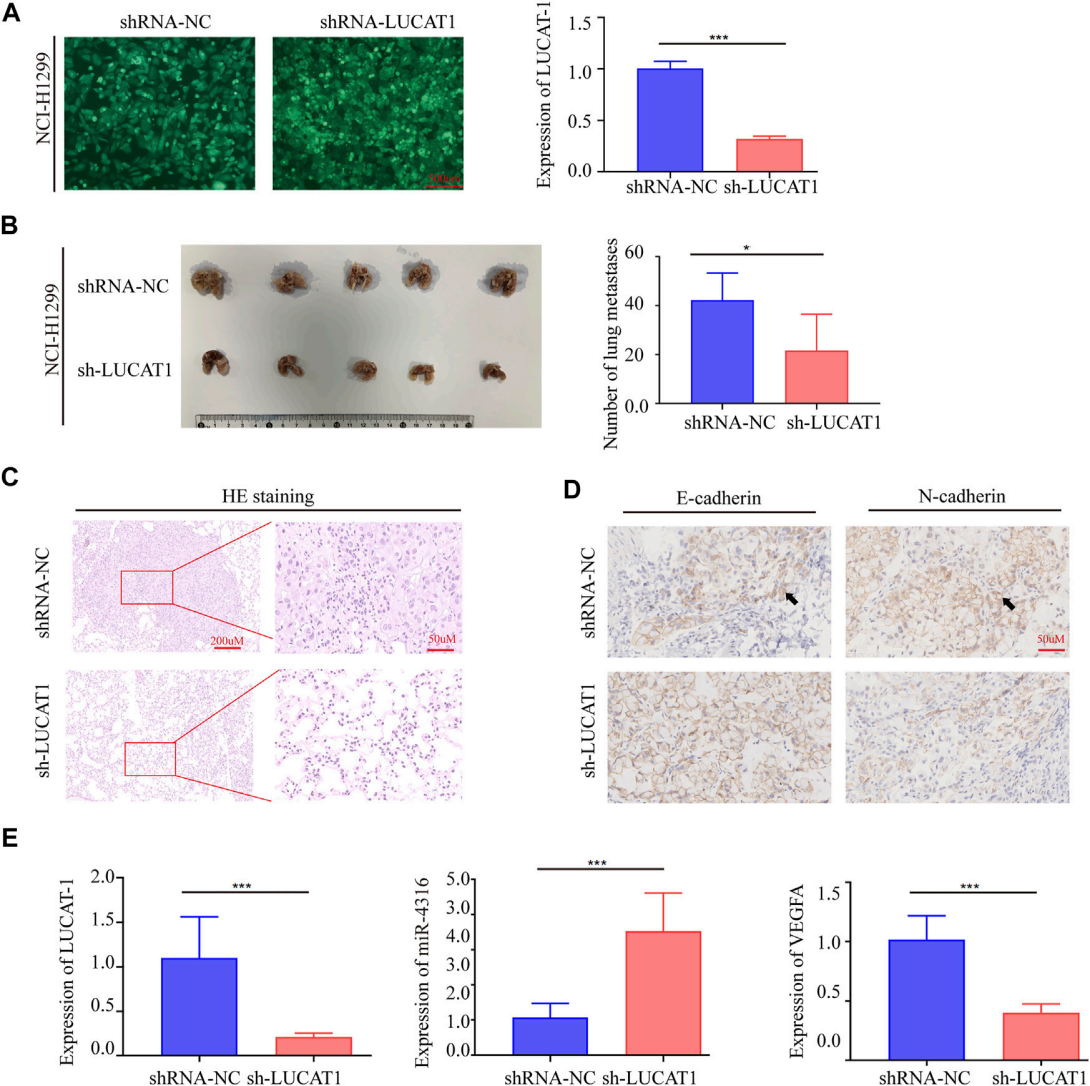
Silencing LUCAT1 inhibits tumor metastasis *in vivo*. **(A)** qRT-PCR results for knockdown efficiency of LUCAT1 in A549 cells by shRNAs. Scale bar: 200 µm. **(B)** Number of metastatic nodules in the lung tissues. **(C)** The microscopic images of lung tissue sections stained by HE. **(D)** IHC were performed to quantity the expression of E-Cadherin and N-Cadherin in metastatic nodes of mouse model. **(E)** qRT-PCR was performed to detect the expression of LUCAT1, miR-4316 and VEGFA of metastatic nodes formed from sh-LUCAT stable cells. *p*-values were determined by two-tailed student’s t-test (*p < 0.05; **p < 0.01; ***p < 0.001).

To further investigate the biological function of LUCAT1, we established *in vivo* xenograft nude mouse model of LUAD metastasis *via* a tail vin injection method. Short hairpin RNA (shRNA) was utilized to stably silence LUCAT1 expression in NCI-H1299 cell lines (Figure 4A). In line with the *in vitro* data, the numbers of metastatic nodules in the lungs were significantly decreased in nude mouse injected with NCI-H1299 sh-LUCAT1 cells compared those injected with NCI-H1299 sh-NC cells (Figure 4B). Metastatic nodules were dissected out for haematoxylin and eosin (HE) analysis and further confirmed the metastasis nodules (Figure 4C). Immunohistochemistry (IHC) was performed to quantity the expression of biomarkers of metastasis in mouse model. Consistent with our observations *in vitro*, silencing of LUCAT1 significantly enhanced E-cadherin expression and attenuated the expression of N-Cadherin in xenografts, suggesting that silencing of LUCAT1 inhibited lung metastasis (Figure 4D). And the expression levels of LUCAT1/miR-4316/VEGF-A axis in metastatic LUAD in mouse model were also investigated *via* qRT-PCR. As expected, metastatic nodes formed from NCI-H1299 sh-LUCAT1 cells exhibited decreased LUCAT1 expression, while the expression of miR-4316 were upregulated along with lung metastasis. Similarly, a significant difference was also observed in the expression of VEGFA between NCI-H1299 sh-LUCAT1 and sh-NC groups in metastatic nodules (Figure 4E). These data showed that LUCAT1 silencing inhibited tumor metastasis *in vivo*.

### Lung Cancer-Associated Transcript 1 Bound Directly to miR-4316 and Negatively Regulated the Expression of miR-4316

**FIGURE 5:**
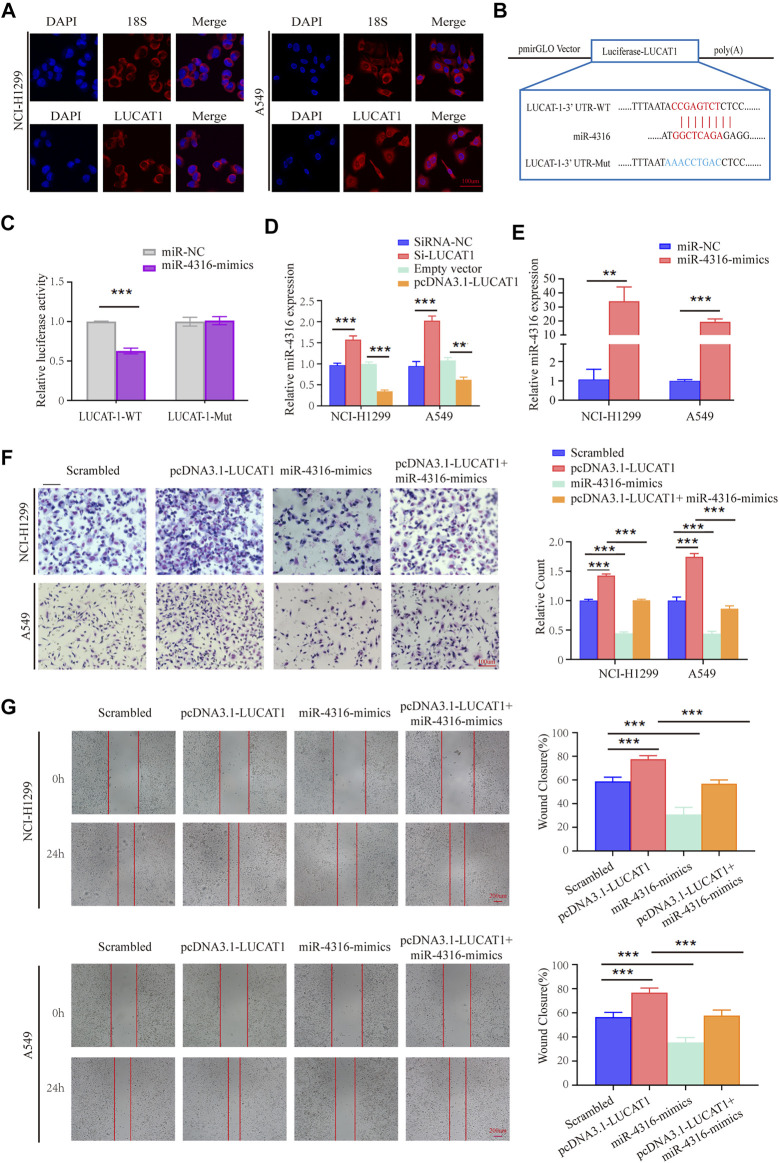
LUCAT1 binds directly to miR-4316 and negatively regulates the expression of miR-4316. **(A)** Subcellular localization of LUCAT1 by RNA FISH. Red fluorescent probe: LUCAT1 and 18s (Cy3 labelled probes); blue fluorescent probe: DAPI. 18s served as a positive control. Scale bar: 100 µm. **(B)** Schematic diagram of the potential binding sites between LUCAT1 and miR-4316. **(C)** The luciferase activity was tested after co-transfection with wild type (WT)/mutant 3′UTR of LUCAT1 and miR-4316 mimics for 48 h. **(D)** Relative expression of miR-4316 in LUAD cells transfected with si-LUCAT, pcDNA3.1- LUCAT1 or control. **(E)** qRT-PCR was performed to detect the expression of miR-4316 in LUAD cells after transfection of miR-4316 mimics, or control. **(F,G)** Migration and invasion ability of LUAD cells co-transfected with negative control mimics, miR-4316 mimics, or/and overexpression plasmid (pcDNA3.1- LUCAT1) were determined by transwell and wound healing assays. *p*-values were calculated using two-tailed student’s t-test (*p < 0.05; **p < 0.01; ***p < 0.001).

To explore whether LUCAT1 promotes LUAD metastasis *via* the competing endogenous RNAs (ceRNAs) mechanism. RNA fluorescence *in situ* hybridization (FISH) assay was performed using an LUCAT1 special probe, and the results revealed that LUCAT1 was mainly localized in the cytoplasm of NCI-H1299 and A549 cells (Figure 5A).

The putative candidate miRNAs binding to LUCAT1 were predicted using the bioinformatics tool miRDB (http://mirdb.org/), and miR-4316 was predicted to interact with LUCAT1. Then, we constructed a reporter vector in which the putative miR-4316-3p-binding site in the LUCAT1 sequence was mutated by base mutations, followed by co-transfection with control miRNA and miR-4316 mimics into HEK293T cells (Figure 5B). As expected, dual-luciferase reporter assay revealed that miR-4316 mimic reduced luciferase activities with the LUCAT1-WT expression vector, but not the LUCAT1-MUT, which suggested that miR-4316 could directly bind to LUCAT1 (Figure 5C). Besides, the regulation of miR-4316 by LUCAT1 in NCI-H1299 and A549 cells were analyzed, and the results showed that knockdown of LUCAT1 significantly promoted the expression of miR-4316. In contrast, the levels of miR-4316 were significantly reduced by LUCAT1 overexpression (Figure 5D). These findings indicated that LUCAT1 targeted directly miR-4316 and negatively regulated its expression.

*In vitro* rescue assays were performed to identify the biological function of LUCAT1/miR-4316 regulatory axis. NCI-H1299 and A549 cells were co-transfected with pcDNA3.1-LUCAT1 and miR-4316 mimics, the transfection efficiency of miR-4316 mimics were determined by qRT-PCR analysis (Figure 5E). The results of the transwell invasion assays showed that overexpression of miR-4316 significantly inhibited cell invasion, and rescued the increased cell invasive capability caused by LUCAT1 overexpression in NCI-H1299 and A549 cells (Figure 5F). Meanwhile, wound healing assays showed the consistent results (Figure 5G), indicating that LUCAT1 attenuating LUAD cells invasion and migration by serving as miR-4316 sponge.

### VEGFA Was a Direct Target of miR-4316 and Regulated by Lung Cancer-Associated Transcript 1

**FIGURE 6:**
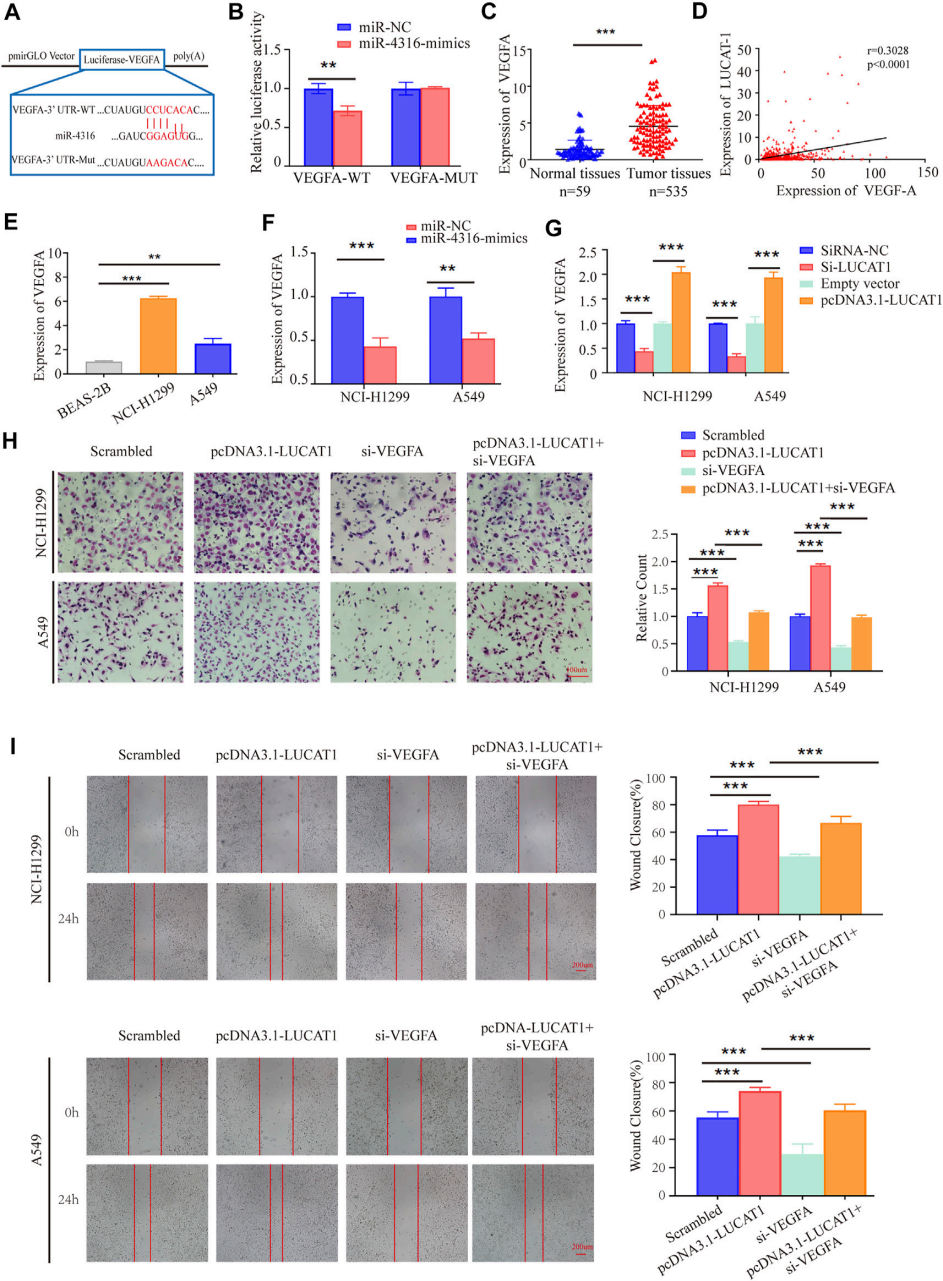
LUCAT1 promotes migration and invasion of LUAD cells *via* LUCAT1/miR-4316/VEGFA axis. **(A)** Schematic representation of WT- and Mut-VEGFA sequences. Red fonts represented the mutant bases. **(B)** The luciferase reporter plasmid containing WT- and Mut-VEGFA was co-transfected into HEK293T cells with miR-4316 or corresponding control. **(C)** VEGFA expression level in LUAD tissues (*n* = 535) and normal tissues (*n* = 59) analysed using the TCGA database. **(D)** Relationship between LUCAT1 and VEGFA was measured using the TCGA database. **(E)** VEGFA expression was measured in normal lung epithelial cell line (BEAS-2B) and established LUAD cell lines (NCI-H1299 and A549) using qRT-PCR. **(F)** qRT-PCR was performed to detect the expression of VEGFA in LUAD cells after transfection of miR-4316 mimics, or control. **(G)** Relative expression of VEGFA in LUAD cells transfected with si-LUCAT, pcDNA3.1- LUCAT1 or control. **(H,I)** Migration and invasion ability of LUAD cells after co-transfection with negative control siRNA, si-VEGFA, or/and overexpression plasmid (pcDNA3.1- LUCAT1) were determined by transwell and wound healing assays. *p*-values were determined by two-tailed student’s t-test or one-way ANOVA (*p < 0.05; **p < 0.01; ***p < 0.001).

MiRNAs have been reported to exert their multiple biological functions mainly through degrading their downstream mRNA. By searching with the online prediction software Target Scan (http://www.targetscan.org/vert_72/), we identified that VEGFA was a potential target of miR-4316. Also, dual-luciferase reporter assay was performed by generating wild-type or WT sequence of VEGFA (Figure 6A). The results showed a significant reduction in luciferase activities after co-transfection of miR-4316 mimic and VEGFA-WT, which provided evidence that miR-4316 could directly bind to VEGFA (Figure 6B).

VEGFA has been proved to promote the metastasis of various types of solid cancers, including LUAD. We then analyzed the TCGA data, and found that VEGFA was upregulated in LUAD tissues compared with normal tissues (Figure 6C). Besides, the expression level of LUCAT1 was positively correlated with the expression level of VEGFA in 535 LUAD samples and 59 normal tissues (Figure 6D). Similarly, a higher expression of VEGFA was also observed in our LUAD cell lines (NCI-H1299 and A549) compared with the immortalized lung epithelial cell line BEAS-2B (Figure 6E).

To determine whether VEGFA was regulated by miR-4316 in LUAD cells, mRNA of VEGFA were measured when miR-4316 was overexpressed in NCI-H1299 and A549 cells. The results indicated that VEGFA were significantly decreased by miR-4316 mimics (Figure 6F). Then, expression levels of VEGFA were also measured when LUCAT1 was overexpressed or knocked down to identify the ceRNA network between LUCAT1 and VEGFA. Results showed that transfection of si-LUCAT1 into NCI-H1299 and A549 cells decreased the mRNA levels of VEGFA *via* releasing miR-4316, and the opposite results were observed when LUCAT1 was upregulated (Figure 6G).

### Lung Cancer-Associated Transcript 1/miR-4316/VEGF-A Axis Was Involved in Lung Adenocarcinoma Progression

To identify the effect of LUCAT1/miR-4316/VEGF-A axis on the biological function in LUAD cells, rescue assays were performed. We examined cells co-transfected with pcDNA3.1- LUCAT1 and si-VEGFA, and found that the enhanced invasion capabilities induced by LUCAT1 overexpression were partially reversed by co-transfection with si-VEGFA in NCI-H1299 and A549 cells (Figure 6H). Wound healing assays showed that si-VEGFA also partially reversed the enhanced migration capability induced by LUCAT1 overexpression (Figure 6I). These results suggested that LUCAT1 upregulated VEGFA expression by acting as miR-4316 sponge, thereby attenuating invasion and migration of LUAD cells.

### Lung Cancer-Associated Transcript 1 Regulated Lung Adenocarcinoma Cell Glycolysis *via* miR-4316/VEGF-A Axis

Cancer cells frequently alter their metabolic pathways to adapt to environmental challenges or facilitate tumor proliferation, metastasis (Feng et al., 2020; Yang et al., 2020). Recently, growing evidence have provided for cancer cells’ dependent on glycolysis for energy production (Vander Heiden et al., 2009). It was reported that LUCAT1 was upregulated under high-glucose conditions, and knockdown of LUCAT1 inhibited the EMT in HG-treated HK-2 cells (Zhang et al., 2020), however, whether LUCAT1 participate in glycolysis of LUAD cells needs to be confirmed.

**FIGURE 7:**
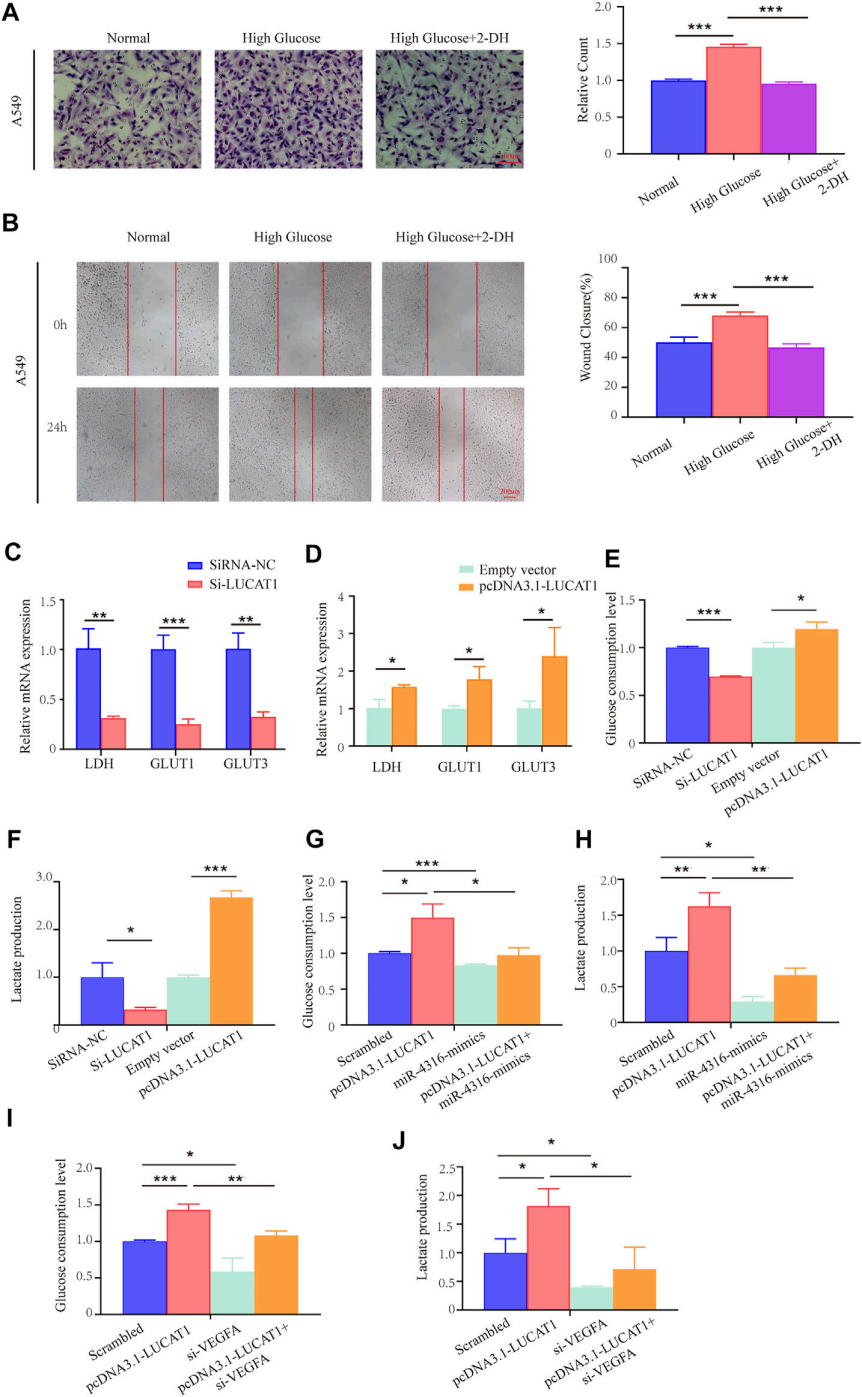
LUCAT1 promotes glycolysis in LUAD cells through the LUCAT1/miR-4316/VEGFA axis. **(A,B)** Migration and invasion ability of LUAD cells were analyzed under normal culture conditions with high concentration of glucose; glycolysis inhibitor 2-DG abolished the ability of the migration and invasion ability of LUAD cells. **(C,D)** qRT-PCR was performed to detect the expression of Glycolysis-related enzyme markers in A549 cells after transfected with si-LUCAT1, pcDNA3.1- LUCAT1 or corresponding negative control. **(E,F)** Lactate production and glucose consumption level were measured after transfected with si-LUCAT1, pcDNA3.1- LUCAT1 or corresponding negative control. **(G,H)** Lactate production and glucose consumption level were measured after co-transfection with negative control mimics, miR-4316 mimics, or/and overexpression plasmid (pcDNA3.1- LUCAT1), using reagent kit according to the instruction of the manufacturers, respectively. **(I,J)** Lactate production and glucose consumption level were measured after co-transfection with negative control siRNA, si-LUCAT1, or/and overexpression plasmid (pcDNA3.1- LUCAT1), using reagent kit according to the instruction of the manufacturers, respectively. *p*-values were calculated by two-tailed student’s t-test or one-way ANOVA (*p < 0.05; **p < 0.01; ***p < 0.001).

We first set out to evaluate the potential influence of high glucose on invasion and migration in LUAD cells. Capabilities of cell’s invasion and migration were determined under normal culture conditions or with high concentration of glucose (Normal glucose: 5.5 mM glucose, High glucose: 33 mM glucose). Our data clearly showed that high glucose culture significantly promoted invasion and migration of A549 cells, in comparison with the normal culture medium (Figures 7A,B). However, when treating with 2-deoxyglucose (2-DG, 0.5 mM), an inhibitor of glucose metabolism and inhibits glycolysis by acting on hexokinase, we found that the high glucose-induced invasion and migration could be effectively reversed (Figures 7A,B).

We also detected metabolic activities and found that silencing LUCAT1 expression in LUAD cells significantly suppressed the expression of metabolic enzymes and transporters, including LDH, GLUT1, GLUT3 (Figure 7C). Conversely, LUCAT1 overexpression promoted their expressions in A549 cells (Figure 7D). To further determine whether LUCAT1 was associated with the glucose metabolic changes in A549 cells, glucose consumption and metabolites were analyzed. As shown in Figures 7E,F, knockdown of LUCAT1 led to a significant decrease in glucose consumptions and lactate productions in A549 cells. In contrast, overexpression of LUCAT1 produced the opposite effects on them in A549 cells. In addition, LUCAT1 overexpression could promote the glucose consumption and lactate productions of A549 cells, while treatment with miR-4316 mimics partially reversed the phenotypes caused by LUCAT1 overexpression (Figures 7G,I). It was consistent with our suspect that knockdown of VEGFA partially reversed the increased glucose consumption and lactate productions induced by LUCAT1 overexpression in A549 cells (Figures 7H,J). These results suggested that LUCAT1 promoted glycolysis of LUAD cells *via* the upregulation of VEGFA by acting as miR-4316 sponge.

## Discussion

Multiple factors were involved in tumorigenesis, and extensive efforts have been devoted to investigating the molecular and cellular mechanisms of LUAD progression, but potential molecular targets for effective antimetastatic therapies remain to be elucidated. Accumulating evidence suggested that lncRNAs were recognized as important regulators of gene expression involved in malignant processes, or directly participated in cancer initiation and progression. LUCAT1 was first known to render lung cancer and associated with poor survival outcomes of patients with smoking-related non-small cell lung cancer (Sun et al., 2017). Further evidence confirmed that LUCAT1 participated in the regulation of multiple processes of tumor occurrence and development (Shen et al., 2020; Wu et al., 2020; Xing et al., 2021), while the detailed role of LUCAT1 in LUAD metastasis and glycolysis was not well known. Our study clarified the specific mechanism of the promotion of LUAD metastasis and glycolysis by lncRNA LUCAT1 through positively regulating VEGFA and sponging miR-4316, which help provide a better understanding of lncRNA-regulated glycolysis, metastasis and LUAD progression.

With the characteristics of noninvasiveness and stable in various body fluids, such as plasma and serum, lncRNA could be utilized as a promising and sensitive biomarker for cancer diagnosis and prognosis (Huarte, 2015; Huang et al., 2020). Evidence has revealed that exosomes provided a relatively stable environment for its contents, and exosomal lncRNAs had advantages of becoming non-invasive biomarkers to distinguish tumor patients and healthy individuals (Mohankumar and Patel, 2016; Liu et al., 2021). In our study, we demonstrated that serum exosomal LUCAT1 was highly expressed in LUAD patients compared with healthy controls based on our high-throughput sequencing and series of validation. ROC analysis further confirmed that serum exosomal LUCAT1 had prominent value in LUCAT1 diagnosis as a blood-based biomarker.

To address the significance of LUCAT1 in pathogenesis of LUAD, we demonstrated that the levels of LUCAT1 were significantly elevated in the tissues of LUAD patients in contrast to adjacent normal tissues. High expression of LUCAT1 was positively correlated with TNM stage, lymph node metastasis and unfavorable prognosis in patients with LUAD. Since whether LUCAT1 was associated with the metastasis and glycolysis of LUAD cells remains unknown, we mainly explored the functions of LUCAT1 in these two aspects in the following research. In our study, LUCAT1 knockdown can hinder the invasion and migration activity of LUAD cells *in vitro* and inhibit tumor metastasis *in vivo*. All these findings elucidated the oncogenic function of LUCAT1 in LUAD.

Previous studies indicated that lncRNAs located in cytoplasm could function as ceRNA *via* competitively binding to miRNAs, thereby positively regulating the downstream target mRNAs (Hirata et al., 2015; Zhu et al., 2020). You and colleagues reported that oncogenic Linc00284 markedly attenuated the expression level of c-Met by binding miR-27a, thereby promoting colorectal cancer cell proliferation and invasion (You et al., 2021). Zheng et al. found that LncRNA FAM225A affected the expression of ITGB3 by disrupting the binding sites for miR-590-3p/miR-1275, thereby contributing to the tumorigenesis and progression of *nocardia* gastric cancer (Zheng Z.-Q. et al., 2019). After having validated the subcellular location of LUCAT1, we verified miR-4316 as potential target of LUCAT1. Meanwhile, luciferase reporter also verified the endogenous interaction between LUCAT1 and miR-4316. Previous studies have shown that miR-4316 might serve as a tumor suppressor and was downregulated in a variety of cancers, including gastric cancer, hepatocellular carcinoma, and bladder cancer (Wang et al., 2018; Changyong et al., 2019). However, the role of miR-4316 in LUAD still unclear. Here, a negative correlation between the expression of LUCAT1 and miR-4316 was observed in LUAD cells. LUCAT1 acting as a miR-4316 sponge to promote LUAD cells proliferation and invasion. The classic mechanism for miRNA was modulate target genes by binding on its complementary binding sites of mRNA 3′-UTR, inhibiting target mRNA translating or promoting mRNA degradation. In this study, we confirmed that VEGFA may serve as a functional target of miR-4316, which has been proved to have inhibitory effects on proliferation and migration (Mousa et al., 2020). Therefore, we suspected that VEGFA is one of the miR-4316 target genes in LUAD cells.

It is shown that VEGFA, as an oncogene, was upregulated in a variety of cancers, including LUAD, and promotes the initiation, development, and poor prognosis of cancer (Liu et al., 2017; Liang et al., 2019; Zhao et al., 2019; Zou et al., 2020). Consistent with these findings, our study demonstrated that VEGFA was increased in LUAD tissues and cell lines, and the interaction between miR-4316 and the 3′UTR of VEGFA mRNA was then validated. Meanwhile, Pearson analysis showed that there was a negative correlation between the expression of miR-4316 and LUCAT1 in LUAD tissue samples. In addition, VEGFA was suppressed by miR-4316 and increased upon LUCAT1 overexpression. Importantly, the co-transfection experiments proved that changes in the levels of LUCAT1, miR-4316 and VEGFA are related to the migration and invasion of LUAD cells, implying that LUCAT1/miR-4316/VEGFA axis participated in LUAD progression. Based on these findings, we concluded that a comprehensive analysis of expression levels of LUCAT1, miR-4316 and VEGFA may help us identify patients at high risk of poor prognosis, and providing useful information of clinical management for them. However, given the complexity of tumor microenvironment, there exist other mechanisms involved in progress of LUAD, which required further investigation.

Even though growing evidence have shown that glycolysis plays an important role in the growth and metastasis of multiple solid cancers (Hu et al., 2019; Yang et al., 2020), the complex regulatory mechanisms of LUAD glycolysis remain elusive. This study proved that high glucose conditions could enhance the ability of migration and invasion in LUAC cells. Lin and colleagues found that LUCAT1 was upregulated under high-glucose conditions, and knockdown of LUCAT1 inhibited the EMT in HG-treated HK-2 cells (Zhang et al., 2020). Therefore, we assumed that LUCAT1 might regulate migration and invasion of LUAD cells by participating in the process of glucose metabolism. Interestingly, we provided evidence that overexpression of LUCAT promoted the glucose consumption and lactate production, and rescue experiments indicated that the enhanced glucose consumption and lactate productions could be reversed by miR-4316 mimics or si-VEGFA. Taken together, our results suggested that LUCAT1 may promote glycolysis of LUAD cells by acting as a ceRNA to sponge miR-4316 and upregulate VEGFA.

Although we have demonstrated that VEGFA silencing could attenuate migration, invasion, glucose consumption and lactate production of cancer cells, a study limitation should be taken into consideration. Since the role of VEGFA in lung cancer was complicated, more independent experimental evidence was needed to support the hypothesizes about how VEGFA regulates metastasis and glucose metabolism. Therefore, more comprehensive studies about the molecular mechanism of VEGFA activating downstream pathway by direct or indirect ways will remain to be conducted in the future.

In summary, our study demonstrated that LUCAT1 was dysregulated in human LUAD serum exosomes and may serve as a potential biomarker in the diagnosis of LUAD. We also identified a novel regulatory mechanism that LUCAT1 could directly interact with miR-4316 in LUAD cells, resulting in increasing VEGFA to promote LUAD cells metastasis and glucose metabolism. These observations complemented the known mechanisms of LUCAT1 in LUAD and indicated a potential target for development of effective therapeutic strategies in LUAD patients.

## Materials and Methods

### Patients and Specimens

A total of 160 LUAD serum samples and 134 healthy serum samples were collected from The Second Hospital of Shandong University between February 2018 and October 2020. All enrolled patients met the following inclusion criteria: primary LUAD was confirmed by histopathology and showed no evidence of other neoplastic diseases in other organs, serum samples were collected before any antitumor therapies, such as surgery, chemotherapy or radiotherapy. The serum samples of controls were prepared from age-matched healthy volunteers. Clinical features of LUAD patients and healthy controls were described in Supplementary Table S1. The tumor stage and grade complied with the 8th Union of International Control of Cancer (UICC) classification.

Fifty LUAD serum samples were randomly selected, mix them thoroughly and divide them into five parts, each of which was regarded as an independent sequencing sample, serum samples of healthy people were also processed in the same way. The remaining samples (including 110 LUAD patients and 84 healthy controls) were selected to verify the expression of LUCAT1 in serum exosomes.

All experiments involved in this study were performed in accordance with relative regulations and manners. All participants have written informed consent and this study was approved by the Clinical Research Ethics Committee of The Second Hospital, Cheeloo College of Medicine, Shandong University [Approval number: KYLL-2019(LW)042].

### Exosome Purification and Identification

Serum isolated from coagulation promoting vacuum tubes within 2 h was immediately transferred to a 1.5 ml Eppendorf tube and immediately centrifuged at 1500 g for 5 min and then 13800 g for 5 min at 4°C to eliminate cell sediments. ExoQuick™ solution (EXOQ5A-1; SBI System Biosciences, United States) was mixed evenly with supernatant according to the manufacturer's instructions. Subsequently, incubate at 4°C for 30 min, followed by centrifuged twice at 4°C (1500 g, 30 min and 1500 g, 5 min), supernatants were discarded, and the exosome pellets were resuspended in 250 μL PBS and stored at −80°C for further analysis.

The morphology of isolated exosomes was imaged using the transmission electron microscopy (TEM; G2 spititi FEI; Tecnai). And the size distribution and concentration of exosomes were quantified by ZETASIZER Nano series-Nano-ZS instrument (Malvern, United Kingdom). Total exosome protein was extracted with RIPA extraction reagent (Thermo Fisher, United States) according to manufacturer’s instructions. Western blotting analysis (WB) were performed to detect the exosomal surface markers: CD9, CD63, and TSG101.

### WB

Proteins from cell lysates were extracted with RIPA buffer (Sigma-Aldrich) according to manufacturer’s instructions, separated by SDS-polyacrylamide gel electrophoresis (SDS-PAGE) and then transferred to Immobilon-P PVDF membrane (Millipore, United States). After being blocked with 5% non-fat milk in TBS-Tween, membranes were incubated with primary specific antibodies and horseradish peroxidase-conjugated secondary antibodies sequentially. Immunoreactive bands were detected using the Clarity Western ECL kit (Bio-Rad) and quantified using Image Lab (Bio-Rad, Hercules, CA, United States). All antibodies used in this study were shown in Supplementary Table S2.

### High Throughput Sequencing

High throughput sequencing service was provided by CloudSeq Biotech (Shanghai, China) and original datasheets could be obtained through access to GEO repository (GSE191209). Briefly, rRNAs were removed from total RNA using Ribo-Zero rRNA Removal Kits (Illumina, United States) according to the manufacturer’s instructions. Subsequently, rRNA-depleted RNAs were used to constructed RNA libraries with TruSeq Stranded Total RNA Library Prep Kit (Illumina, United States). BioAnalyzer 2100 system (Agilent Technologies, United States) was performed to control the quality and quantified. Libraries mentioned above were denatured as single-stranded DNA molecules, after captured on Illumina flow cells, amplified as clusters, and finally sequenced on Illumina HiSeq Sequencer for 150 cycles. The differentially expressed RNAs were selected with log_2_ (fold change) > 1 or log_2_ (fold change) <−1 and with statistical significance (*p* value <0.05) by R package–Deseq2.

### RNA Extraction and Quantitative Real-Time Polymerase Chain Reaction Analysis

Total exosomal RNA was extracted from miRNeasyMicro Kit (Qiagen) according to the manufacturer’s instructions. Concentration and integrity of exosomal RNA were measured by NanoDrop spectrophotometer (Thermo Fisher Scientific). Purified RNA was reversely transcribed into cDNA using the PrimeScript™ RT reagent kit (Takara, Dalian, Liaoning, China) according to manufacturer’s instructions.

The expression of lncRNA/mRNA was detected by CFX96™ Real-Time System (Bio-Rad Laboratories, Hercules, CA, United States) using Power SYBR Green (Takara, Dalian, China) and GAPDH was selected as the housekeeping gene. The expression of miRNA was measured using primer of miR-4316 and U6 was used as an internal control. All the primer sequences were available in Supplementary Table S3. The relative expressions of lncRNA/mRNA/miRNA were calculated using the 2^−ΔΔct^ method.

### *in situ* Hybridization

The expression level of LUCAT1 in tissues was detected by ISH using a specific digoxigenin-labeled LUCAT1 probe on TMAs, which contained 98 LUAD samples and 82 paired adjacent normal samples. All detailed clinical information (including age, gender, tumor–node–metastasis stage) of these LUAD patients were obtained from Qutdo Biotech (Shanghai, China), and shown in Supplementary Table S4. Further, the quantitative scanning approach was taken to analyse the staining and expression of LUCAT1 using a Nikon microscope. The results of ISH were calculated by multiplying the value of positive staining intensity by the proportion of positively stained cells. In our study, ISH score of <1.0 was regarded as LUCAT1 low-expression group, while ≥1.0 indicated LUCAT1 high-expression. All these experiments were approved by the ethics committee of Shanghai Outdo Biotech Company.

### Cell Lines

Immortalized lung epithelial cell line BEAS-2B and LUAD cell lines (A549, NCI-H1299) were obtained from the Chinese Academy of Sciences Cell Bank (Shanghai, China). Cell lines were cultured with 1640 (Gibco) supplemented with 10% fetal bovine serum (FBS, Australia Origin, Gibco). All cell lines were cultured in a humidified chamber with 5% CO_2_ and 95% air at 37°C.

### Cell Transfections

Si-LUCAT1, miR-4316 mimic, si-VEGFA, si-NC, and mimics-NC were obtained from GenePharma (Shanghai, China). LUCAT1 overexpression plasmids pcDNA3.1-LUCAT1, NC plasmids pcDNA3.1-NC were synthesized by Biosune (Shanghai, China). Lipofectamine™ 2000 was employed to accomplish the cell transfections, following guidelines from the manufacturer. Supplementary Table S5 depicts the sequences of si-RNA and mimics utilized in this study.

### Generation of Stable Cell Lines

To establish stable LUCATI knockdown cells, a lentivirus vector encoding specific sh-LUCAT1 or sh-NC sequences was constructed by Vigenebio (Jinan, China), which could express the green fluorescent protein (GFP) and puromycin resistance gene. After incubated lentivirus vector with the LUAD cells for at least 48 h, fluorescence microscope and RT-qPCR were performed to verify the knockdown efficiency.

### Transwell Assays and Wound Healing Test

Cell migration ability was measured by the transwell chambers (8 μm pore size; Costar), and the ability of cell invasion was evaluated by the transwell chambers with Matrigel (BD Biosciences, San Jose, CA, United States) in the upper chamber. After incubation at 37°C, 5% CO_2_ for 24 h, the lower chambers were fixed in methanol, stained with 0.1% crystal violet, and imaged by Inversion Microscope (Zeiss, Germany). Cell numbers for cell invasion in three random fields were counted.

For wound healing assays, cells were seeded into 6-well plates, and a 200 μL pipette tip was used to create an artificial scratch when incubated to 100% confluence. Subsequently, the cells were cultured in serum-free medium, wound closure images were captured in the same field at 0 and 24 h. The fraction of cell coverage across the line were used to calculate the cell healing rates.

### Animal Experiments

For the *in vivo* tumor metastasis experiments, BALB/c nude mice were divided into two groups (*n* = 5) to establish the lung metastasis model. 1 × 10^6^ NCI-H1299 cells stably transfected with sh-LUCAT1 or shRNA-NC in 0.1 ml PBS were subcutaneously injected into the tail vein of the mice above. Six weeks after injection, the mice were euthanatized and dissected. Lungs of the mice in two groups were removed and photographed, metastatic nodules in lungs were counted by naked eye and investigators were blinded to the group allocation. The criteria used to identify metastatic nodules were as follows: whitish and uniformly colored, round, more than 1 mm in diameter, and the appearance quality was different from the surrounding normal tissue. After tissue were fixed in paraformaldehyde, embedded in paraffin, hematoxylin and eosin (HE) staining were performed to confirm the metastatic nodes. The animal studies were carried out in accordance with NIH Guidelines for the Care and Use of Laboratory Animals and approved by the Animal Care Committee of The Second Hospital, Cheeloo College of Medicine, Shandong University. The ethics approval number of Animal experiments was KYLL-2019(LW)043.

### RNA Fluorescence *in situ* Hybridization

Cy3-labeled LUCAT1 and U6 sense probe were obtained from GenePharma (Shanghai, China). Fluorescence processed RNA FISH assay *in Situ* Hybridization Kit (GenePharma, Shanghai, China) were performed according to the manufacturer's instructions. Briefly, NCI-H1299 and A549 cells were washed, fixed and treated by 0.1% Triton X-100. After incubated with labelled FISH probe pre-mixed solution overnight in the dark, 4,6-diamidino-2-phenylindole (DAPI) (Solarbio, Beijing, China) was used to stain the cell nucleus for 10 min. Finally, fluorescence images were produced by a confocal microscope (Carl Zeiss Microscopy, LLC, United States). All the probe sequences used in this study were available in Supplementary Table S6.

### Luciferase Reporter Assay

For luciferase reporter assays, we constructed wild-type (WT) LUCAT1/VEGFA reporter plasmid in which the putative miR-4316-binding site in the LUCAT1sequence, and mutated-type (MUT) LUCAT1/VEGFA reporter plasmid in which the putative miR-4316-binding site in the LUCAT1sequence was mutated by base mutations with pmirGLO promoter vector. Luciferase reporter vectors were stably co-transfected with miRNA-4316 mimic or mimic-NC by Lipofectamine™ 2000 Transfection Reagent (Invitrogen, United States) according to the manufacturer’s guidelines. Luciferase intensity was measured using a dual-luciferase reagent (Promega) according to the manufacturer’s instructions. Renilla luciferase intensity was used as the control. Sequence of luciferase reporter associated with this article were listed in Supplementary Table S7.

### Glucose Consumption and Lactate Production Assays

A549 cells were seeded into six-well plates at a density of 1 × 10^6^ cells/well and cultured overnight. Glucose assay kits (Solarbio) and lactate assay kits (Solarbio) were used to detect the glucose consumption and lactate production in A549 cells, according to the manufacturer’s instructions, respectively. All data were normalized by the cell numbers.

### Glucose Conditions

A549 cells were cultured in RPMI-1640 medium (Gibco, Shanghai, China) supplemented with 10% fatal bovine serum (FBS) (Sagecreation, Beijing, China). After reaching 70–80% confluence, A549 cells were washed and then growth arrested in serum-free RPMI-1640 medium for 24 h to synchronize the cell growth. Then, d-glucose (Solarbio, Beijing, China) at a final concentration of 33mM, or 2-Deoxy-D-glucose (2-DG) (Solarbio) at a concentration of 0.5 mmol/L were added to the culture medium for an additional 48 h until further use.

### Statistical Analysis

The experimental data were statistically analysed using Graph Pad Prism 6.0 and SPSS 13.0 software. The mean values of two groups were assessed by Student’s t test or the Mann-Whitney U test as appropriate. Kaplan–Meier plots and log-rank tests were used for the survival analysis. The diagnostic performance of LUCAT1 was performed on MEDCALC 15.2.2 (Med-Calc, Belgium). Statistical significance was achieved when *p* values were less than 0.5. All results were expressed as the means ± standard deviation (SD) of three independent experiments.

## Data Availability

The original contributions presented in the study are publicly available. This data can be found here: https://www.ncbi.nlm.nih.gov/, GSE191209.
