# Supplementary material for: Characterization of a Novel LUCAT1/miR-4316/VEGF-A Axis in Metastasis and Glycolysis of Lung Adenocarcinoma
Source: Front Cell Dev Biol. 2022 May 13;10:833579. doi: 10.3389/fcell.2022.833579 (PMC9136330; doi:10.3389/fcell.2022.833579)
Supplement: Supplementary file 1 [file DataSheet1.docx]

Supplementary Tables

**Supplementary Table 1: Characteristics of individuals who provide serums in our study.**

|  | **Variable** | **Sequencing (n=100)** | **Validation (n=194)** |
| --- | --- | --- | --- |
| **Controls (number)** |  | 50 | 84 |
| **Age** | median (IQR), years | 58 (54-62) | 58 (54-65) |
| **Sex (%)** |  |  |  |
|  | Male | 23 (46.00%) | 28 (33.33%) |
|  | Female | 27 (54.00%) | 56 (66.67%) |
| **LUAD (number)** |  | 50 | 110 |
| **Age** | median (IQR), years | 62 (52-67) | 59 (53-66) |
| **Sex (%)** |  |  |  |
|  | Male | 22 (44.00%) | 35 (31.82%) |
|  | Female | 28 (56.00%) | 75 (68.18%) |
| **Location (%)** |  |  |  |
|  | Left | 15 (30.00%) | 35 (31.82%) |
|  | Right | 35 (70.00%) | 75 (68.18%) |
| **Size (%)** |  |  |  |
|  | ＜ 2 cm | 25 (50.00%) | 53 (48.18%) |
|  | ≥ 2 cm | 25 (50.00%) | 57 (51.82%) |
| **LNM (%)** |  |  |  |
|  | Negative | 44 (88.00%) | 88 (80.00%) |
|  | Positive | 6 (12.00%) | 22 (20.00%) |
| **TNM stage (%)** |  |  |  |
|  | I-II | 48 (96.00%) | 94 (85.45%) |
|  | III-IV | 2 (4.00%) | 16 (14.55%) |

Abbreviations: LUAD: Lung adenocarcinoma, LNM: Lymph node metastasis

**Supplementary Table 2: All antibodies used in this study.**

| **Antibody** | **Item No./** **Manufacturer** |
| --- | --- |
| anti-CD9 antibody (rabbit IgG) | 13174S/CST, USA |
| anti-TSG101 (mouse IgG) | Ab83/ Abcam, UK |
| Anti-CD63 (rabbit IgG) | ab134045/ Abcam, UK |
| goat anti-rabbit HRP secondary antibody | ZB-2301/ ZSGB-BIO, China |
| goat anti-mouse HRP secondary antibody | ZB-2305/ZSGB-BIO, China |
| E-Cadherin (rabbit IgG) | 3195S/CST, USA |
| N-Cadherin (rabbit IgG) | 13116S/CST, USA |
| β-Catenin (rabbit IgG) | GB11015/servicebio, China |
| vimentin (mouse IgG) | ab8978/ Abcam, UK |
| GAPDH (rabbit IgG) | 5174S/CST, USA |

**Supplementary Table 3: Primer sequences.**

| **Gene name** | **Primer sequences** |
| --- | --- |
| **ENST00000417930** | Forward primer 5’- TGGAGTCACACTACCAAAGGC-3’  Reverse primer 5’- GCAAGAGAGACAGATCGTCCA-3’ |
| **uc010jub.1** | Forward primer 5’- TGAGAAACCAGCCAGCAAGT -3’  Reverse primer 5’- TTGAAGTCTCTGGAGTGAAACA-3’ |
| **ENST00000513626** | Forward primer 5’- AACCATGTGTCAAGCTCGGAT -3’  Reverse primer 5’- GTGCCAAGGTCCCATAAGAGT -3’ |
| **GAPDH** | Forward primer 5’- ACCCACTCCTCCACCTTTGAC -3’  Reverse primer 5’- TGTTGCTGTAGCCAAATTCGTT -3’ |
| **VEGFA** | Forward primer 5’-GCAGAATCATCACGAAGTGGT -3’  Reverse primer 5’- CCAGGGTCTCGATTGGATGG-3’ |
| **MiR-4316** | Primer 5’- TATGGTGAGGCTAGCTGGTG-3’ |
| **U6** | Forward primer 5’-CTCGCTTCGGCAGCACA -3’  Reverse primer 5’- AACGCTTCACGAATTTGCGT-3’ |
| **LDH** | Forward primer 5’- TTGTTGGGGTTGGTGCTGTTG -3’  Reverse primer 5’- AAGAGCAAGTTCATCTGCCAAG -3’ |
| **GLUT1** | Forward primer 5’- GAGCAGCTACCCTGGATGTC -3’  Reverse primer 5’- GGAAGCACATGCCCACAATG -3’ |
| **GLUT3** | Forward primer 5’- CCGCTGCTACTGGGTTTTAC -3’  Reverse primer 5’- ACCGCTGGAGGATCTGCTTA -3’ |

**Supplementary Table 4: The correlation between the expression of LUCAT1 and clinical-pathological characterizations of LUAD patients in ISH**

|  | **variables** | **LUCAT1 expression** | | **total** | **χ^2^** | ***p* value** |
| --- | --- | --- | --- | --- | --- | --- |
|  |  | **Low** | **High** |  |  |  |
| **Age (year)** |  |  |  |  | 0.713 | 0.398 |
|  | ≤66 | 36 | 34 | 70 |  |  |
|  | >66 | 13 | 8 | 21 |  |  |
| **Sex** |  |  |  |  | 0.207 | 0.649 |
|  | Female | 21 | 20 | 41 |  |  |
|  | male | 28 | 22 | 50 |  |  |
| [**Tumor**](D:/Program%20Files%20(x86)/Youdao/Dict/8.9.6.0/resultui/html/index.html#/javascript:;) [**size**](D:/Program%20Files%20(x86)/Youdao/Dict/8.9.6.0/resultui/html/index.html#/javascript:;) |  |  |  |  | 0.807 | 0.369 |
|  | ≤5cm | 40 | 31 | 71 |  |  |
|  | >5cm | 9 | 11 | 20 |  |  |
| **Grade** |  |  |  |  | 1.269 | 0.26 |
|  | I/II | 34 | 25 | 59 |  |  |
|  | III | 14 | 17 | 31 |  |  |
| **TNM stage** |  |  |  |  | 12.312 | <0.0001 |
|  | Ι/II | 40 | 19 | 59 |  |  |
|  | III/IV | 9 | 22 | 31 |  |  |
| **T stage** |  |  |  |  | 5.002 | 0.025 |
|  | T1/T2 | 39 | 26 | 65 |  |  |
|  | T3/T4 | 8 | 16 | 24 |  |  |
| **N stage** |  |  |  |  | 1.767 | 0.184 |
|  | N0 | 26 | 16 | 42 |  |  |
|  | N1/N2/N3/Nx | 23 | 25 | 48 |  |  |
| **M stage** |  |  |  |  | 1.180 | 0.277 |
|  | M0 | 49 | 41 | 90 |  |  |
|  | M1 | 0 | 1 | 1 |  |  |

**Supplementary Table 5: Sequence of siRNA and mimics associated with this article.**

|  |  |  |  |
| --- | --- | --- | --- |
| **Si-RNA** | **Negative control** | **Sense** | 5’-UUCUCCGAACGUGUCACGUTT-3’ |
|  |  | **Antisense** | 5’-ACGUGACACGUUCGGAGAATT-3’ |
|  | **LUCAT1-homo-468** | **Sense** | 5’-CCAACUUGCUGUUUGCUAUTT-3’ |
|  |  | **Antisense** | 5’-AUAGCAAACAGCAAGUUGGTT-3’ |
|  | **VEGFA-homo-150** | **Sense** | 5’-GGCAGAAUCAUCACGAAGUTT-3’ |
|  |  | **Antisense** | 5’-ACUUCGUGAUGAUUCUGCCTT-3’ |
| **mimics** | **MiR-4316 mimics** | **Sense** | 5’-GGUGAGGCUAGCUGGUG-3’ |
|  |  | **Antisense** | 5’-CCAGCUAGCCUCACCUU-3’ |
|  | **Negative control** | **Sense** | 5’-UUCUCCGAACGUGUCACGUTT-3’ |
|  |  | **Antisense** | 5’-ACGUGACACGUUCGGAGAATT-3’ |

**Supplementary Table 6: Probe sequences used in FISH.**

| **Gene name** | **Primer sequences** |
| --- | --- |
| **LUCAT1** | 5’-CTGGGCAT+TGTCTGAGTGGAGTGT+TGATTCTCTGAACCAAT+TTTGT+TAACGTGAGAAGGAGCCAGAAG+TCAGAACACA+TAG-3’ |
| **18S** | 5’-CTGCCTTCCTTGGATGTGGTAGCCGTTTC-3’ |

**Supplementary table 7:** **sequence of luciferase reporter associated with this article**

| **NAME** | **SEQUENCE** |
| --- | --- |
| **LUCAT1 3'UTR- WT** | 5’-ACATACAATCAACACTCCACTCAGACAATGCCCAGACCTCCAGAAACCATGTGTCAAGCTCGGATTGCCTTAGACAGGTGCAATTTAAGAACAGCTTTCATCCTCTTTTCTCTCATATTGTCACACTATGTGTTCTGACTTCTGGCTCCTTTCCTCACAAGAAGCTCACCCAGCTGGAACTCTTATGGGACCTTGGCACCAGAGACCACAAATTCCTCTTTGAAGTTTTCTAACAGCAACAATGGTATTTCTGACTTGGCTTTCTTGTATTTCTCTCACGTTAACAAAATTGGTTCAGCAAGAGGAAAATATAGTTAAATACATTTACATTCCCAGAACTGTTAATATTGAAGAATAAAACACACAGTGGGAAAGAGACGAAGAGAAAATACGCCTGTTTTTATGACTTGTTGATATCTACAGGGTTTTTCCTTAAGCCTCACAACAATTTACAGCATTGCCAGCAGCATTCAGTACATTCAGAACAGGATAATTTCACCCGCAGCTGAACTAGGGCCATGCCTGGGACAGACAGAGAATGGCCTTCCAGCAGACATGCCAAGAAGCAAGCCAGGGTCAGTGAGTGAAGAGGAGGGCAGG-3’ |
| **LUCAT1**  **3'UTR-MUT** | 5’-ACATACAATCAACACTCCACTCAGACAATGCCCAGACCTCCAGAAACCATGTGTCAAGCTCGGATTGCCTTAGACAGGTGCAATTTAAGAACAGCTTTCATCCTCTTTTCTCTCATATTGTCACACTATGTGTTCTGACTTCTGGCTCCTTTAAGACAGAGAAGCTCACCCAGCTGGAACTCTTATGGGACCTTGGCACCAGAGACCACAAATTCCTCTTTGAAGTTTTCTAACAGCAACAATGGTATTTCTGACTTGGCTTTCTTGTATTTCTCTCACGTTAACAAAATTGGTTCAGCAAGAGGAAAATATAGTTAAATACATTTACATTCCCAGAACTGTTAATATTGAAGAATAAAACACACAGTGGGAAAGAGACGAAGAGAAAATACGCCTGTTTTTATGACTTGTTGATATCTACAGGGTTTTTCCTTAATAAGACAGACAATTTACAGCATTGCCAGCAGCATTCAGTACATTCAGAACAGGATAATTTCACCCGCAGCTGAACTAGGGCCATGCCTGGGACAGACAGAGAATGGCCTTCCAGCAGACATGCCAAGAAGCAAGCCAGGGTCAGTGAGTGAAGAGGAGGGCAGG-3’ |
| **VEGFA 3'UTR- WT** | 5’-TTACTCTCACCTGCTTCTGAGTTGCCCAGGAGACCACTGGCAGATGTCCCGGCGAAGAGAAGAGACACATTGTTGGAAGAAGCAGCCCATGACAGCTCCCCTTCCTGGGACTCGCCCTCATCCTCTTCCTGCTCCCCTTCCTGGGGTGCAGCCTAAAAGGACCTATGTCCTCACACCATTGAAACCACTAGTTCTGTCCCCCCAGGAGACCTGGTTGTGTGTGTGTGAGTGGTTGACCTTCCTCCATCCCCTGGTCCTTCCCTTCCCTTCCCGAGGCACAGAGAGACAGGGCAGGATCCA-3’ |
| **VEGFA 3'UTR-MUT** | 5’-TTACTCTCACCTGCTTCTGAGTTGCCCAGGAGACCACTGGCAGATGTCCCGGCGAAGAGAAGAGACACATTGTTGGAAGAAGCAGCCCATGACAGCTCCCCTTCCTGGGACTCGCCCTCATCCTCTTCCTGCTCCCCTTCCTGGGGTGCAGCCAAAAGGACCTATGTAAGACAACCATTGAAACCACTAGTTCTGTCCCCCCAGGAGACCTGGTTGTGTGTGTGTGAGTGGTTGACCTTCCTCCATCCCCTGGTCCTTCCCTTCCCTTCCCGAGGCACAGAGAGACAGGGCAGGATCCA-3’ |
